# Enhancing photosynthesis in plants: the light reactions

**DOI:** 10.1042/EBC20170015

**Published:** 2018-03-21

**Authors:** Tanai Cardona, Shengxi Shao, Peter J. Nixon

**Affiliations:** Department of Life Sciences, Sir Ernst Chain Building – Wolfson Laboratories, Imperial College London, South Kensington Campus, London SW7 2AZ, U.K.

**Keywords:** antenna, biomass, efficiency, photosynthesis, photosystems, photoprotection

## Abstract

In this review, we highlight recent research and current ideas on how to improve the efficiency of the light reactions of photosynthesis in crops. We note that the efficiency of photosynthesis is a balance between how much energy is used for growth and the energy wasted or spent protecting the photosynthetic machinery from photodamage. There are reasons to be optimistic about enhancing photosynthetic efficiency, but many appealing ideas are still on the drawing board. It is envisioned that the crops of the future will be extensively genetically modified to tailor them to specific natural or artificial environmental conditions.

## Improving photosynthesis

In the next three decades, the global demand for food is expected to double [1]. However, crop yield trends are insufficient to meet this demand without tremendous environmental damage [2,3]. While it is likely that significant gains in yield can be achieved by optimizing the global food production system [4,5], an emerging attractive vision to address this problem is the possibility of engineering crops with improved photosynthesis and resilience to environmental stress [6,7].

**Figure 1:**
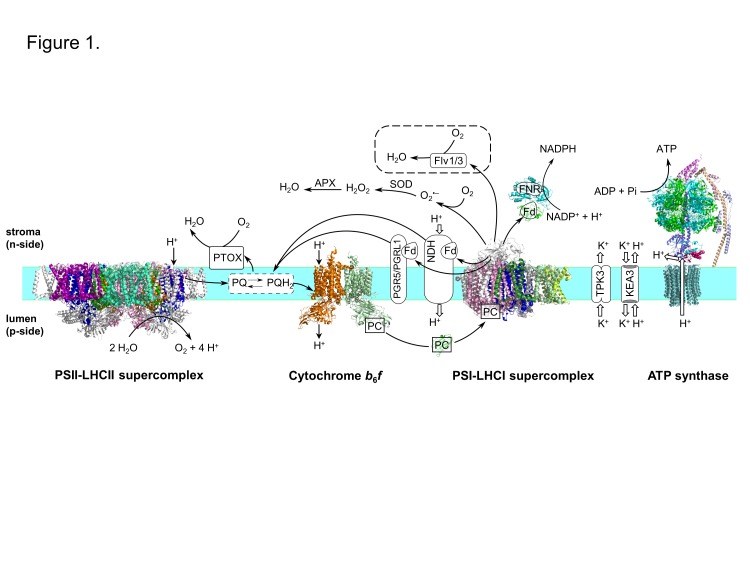
Light reactions of photosynthesis and associated alternative electron transfer pathways Linear electron flow from water to NADP^+^ results in protons being pumped into the lumen of the thylakoid membrane, which is then used to drive the formation of ATP at the ATP synthase. Alternative electron transfer pathways, such as PGR5/PGRL1- and NDH-mediated cyclic electron flow or PTOX- and APX-mediated water-to-water cycles, confer dynamic protection and prevent the formation of reactive oxygen species (ROS). In addition, ion channels and transporters respond to light fluctuations to regulate the proton motive force (*pmf*). All these are potential targets to optimize photosynthetic efficiency. The dashed box indicates a possible non-native route involving Flv1/Flv3 transferred from cyanobacteria to higher plants. Components lacking a high resolution structure are indicated as boxes. Reactions are not balanced. Empty arrows indicate ion movement via channels. Abbreviations: APX, ascorbate peroxidase; Fd, ferredoxin; Flv, flavodiiron protein; FNR, ferredoxin:NADP^+^ reductase; KEA3, potassium efflux antiporter 3; LHC, light-harvesting chlorophyll-a/b-binding complex; NDH, NADH dehydrogenase-like complex; PC, plastocyanin; PQ, plastoquinone; PQH_2_, plastoquinol; PGR5, proton gradient regulation 5; PGRL1, PGR5-like protein 1; PSI, Photosystem I; PSII, Photosystem II; PTOX, plastid terminal oxidase; SOD, superoxide dismutase; TPK3, two-pore potassium channel 3. The following protein structures were used: PSII-LHCII from *Pisum sativum* (PDB ID: 5XNL); cytochrome *b_6_f* from *Chlamydomonas reinhardtii* (PDB ID: 1Q90); PC from *Spinacia oleracea* (PDB ID: 1YLB); Fd-FNR from *Zea mays* (PDB ID: 1GAQ); PSI-LHCI from *Pisum sativum* (PDB ID: 4XK8). The CF_o_F_1_-ATPase is a speculated model based on the yeast F_o_F_1_-ATP synthase (PDB ID: 4B2Q), the chloroplast F_1_-ATPase from *Spinacia oleracea* (PDB ID: 1FX0) and the homo 14-mer c ring of the ATP synthase from *Triticum aestivum* (PDB ID: 4MJN).

The process of photosynthesis has historically been considered to consist of two parts: the light reactions occurring in the thylakoid membrane system that produce ATP and NADPH, see Figure 1; and the light-independent carbon reactions that use ATP and NADPH to fix atmospheric CO_2_ into organic molecules [8].

**Figure 2:**
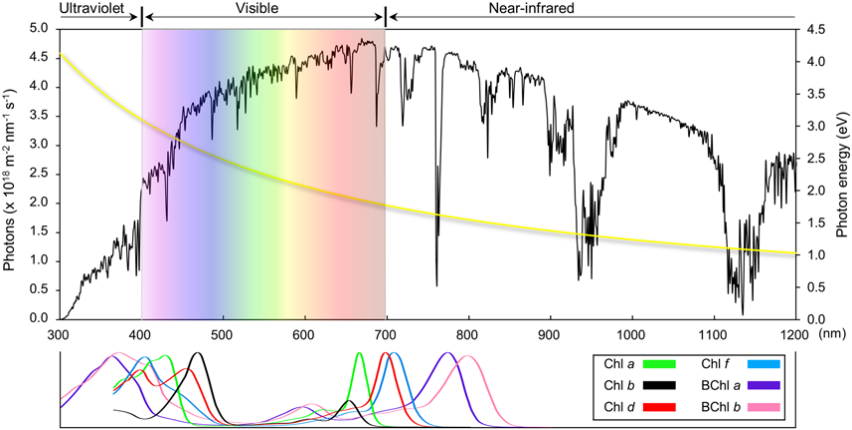
Solar spectrum and absorption profiles of chlorophyll and bacteriochlorophyll pigments The photon flux spectrum from 300 to 1200 nm of sunlight is plotted in black. Original data were obtained from the ‘Standard Tables for Reference Solar Spectral Irradiances: Direct Normal and Hemispherical on 37° Tilted Surface’ (https://www.astm.org/Standards/G173.htm). The UV, visible and near IR regions are indicated. Photon energy at each wavelength is plotted in yellow. Indicative absorption spectra of different chlorophyll and bacteriochlorophyll species in various solvents are normalized to the absorption maxima [22,75].

**Figure 3:**
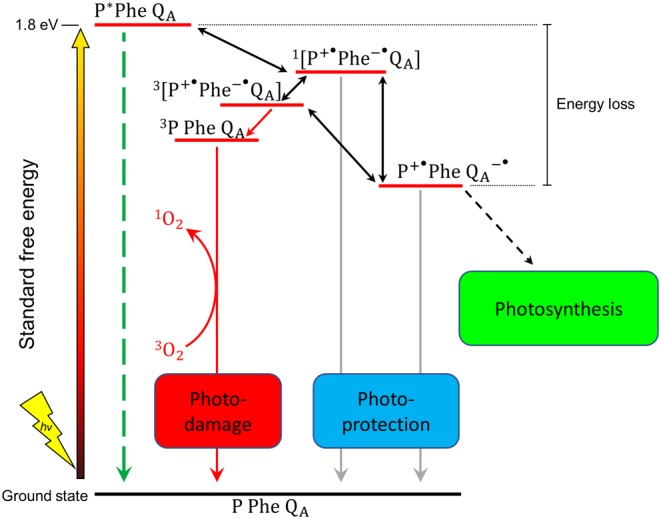
Charge recombination pathways in PSII The energy gaps are not drawn to scale. Safe routes for charge recombination between P^+^ and Q_A_^−^ are indicated in blue, the damaging route producing ^1^O_2_ in red and radiative pathway in green. Stabilization of the P^+^PheoQ_A_^−^ state helps prevent reverse electron flow to form P^+^Pheo^−^Q_A_ and subsequent charge recombination to form PPheoQ_A_. For clarity, the details of the additional electron transfer steps, including the oxidation of water and the reduction of plastoquinone to plastoquinol by PSII, collectively termed as photosynthesis, are omitted. Abbreviations: P, primary electron donor of PSII; Pheo, pheophytin electron acceptor; Q_A_, primary plastoquinone electron acceptor; ^1^O_2_, singlet oxygen; ^3^O_2_, triplet oxygen; ^3^P, triplet excited state of P.

**Figure 4:**
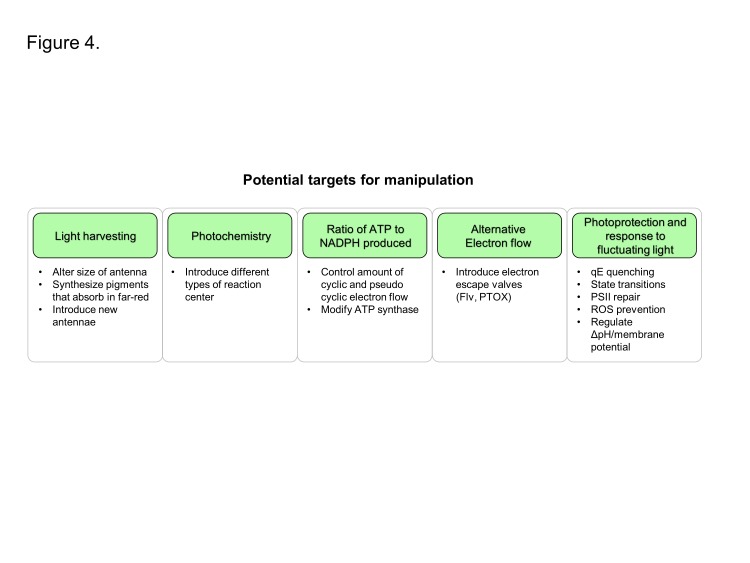
Potential targets for enhancing the light reactions. See text for details.

Overall plant photosynthesis is relatively inefficient. For C3 and C4 photosynthesis, the theoretical maximum efficiency of solar energy conversion into biomass is estimated to be 4.6 and 6% respectively, with much lower efficiencies usually observed in the field [6]. Approximately 75% of the incident solar energy is lost in the initial stages of light collection because not all the light spectrum is used, some of it is reflected or transmitted and some wasted as heat [9]. Some of the losses of energy are intrinsic to the optical properties of the pigments involved in capturing light and are unavoidable, such as the rapid relaxation of higher excited states of chlorophyll generated by absorption of ‘blue’ photons to lower excited states equivalent to the absorption of a ‘red’ photon (Figure 2). Other losses in energy are required to ensure that the rates of forward electron transfer in photosystem I (PSI) and photosystem II (PSII) are much greater than the rates of the back reactions thereby maintaining a high quantum yield of charge separation. Stabilizing electron transfer intermediates in PSII also provides physiologically important thermodynamic barriers to slow down indirect routes of charge recombination that lead to the formation of chlorophyll triplet states and the production of highly reactive singlet oxygen [10] (Figure 3). Despite the fact that some energy loss is inevitable or is important for photoprotection, there are still areas where the efficiency of the light reactions could potentially be improved and result in a significant enhancement of crop yields. Approaches we discuss are: (i) improving light harvesting and photochemistry, (ii) changing the ratio of ATP/NADPH produced by the light reactions, (iii) introducing alternative electron transport pathways and (iv) enhancing photoprotection and the efficiency of electron transport under fluctuating environmental conditions (Figure 4).

## Enhancing light capture

A possible strategy to improve photosynthetic efficiency is to optimize light collection and use. In plants, each photosystem is associated with large antenna systems made of hundreds of chlorophylls and dozens of carotenoids [11,12]. Leaves in the upper parts of the canopy shade those underneath. It is conceivable that reduced antenna systems could allow greater and more uniform light penetration, and therefore enhance photosynthesis [13].

Early attempts focusing on algae have had mixed success [14–16] but more recent work on higher plants appears promising. For example, a mutant of tobacco with reduced antenna was shown to accumulate 25% more stem and leaf biomass in comparison with the wild-type [13]. Recent biophysical models based on measurements of 67 soybean accessions showed that lower amounts of leaf chlorophyll could result in 9% savings in leaf nitrogen without penalties in the rates of photosynthesis [17], but further improvements can be expected [18]. This is due to the fact that soybean seems to over-invest in chlorophyll synthesis [19]. Pot and field experiments using rice with up to 50% less chlorophyll content showed an increase in the rates of photosynthesis of up to 40%, displayed elevated concentrations of ribulose-1,5-bisphosphate carboxylase/oxygenase and faster growth rates, which translated into similar yields to the wild-type in less time [20]. Another mutant of *Arabidopsis thaliana*, possibly affected in the regulation of chlorophyll synthesis, showed improvements in light use, which translated into a 50% increase in the amounts of accumulated glucose and fructose, as well as more than 10% dry-weight biomass in mature plants [21].

Another more radical approach to improving light-energy use is the possibility of engineering crops with light harvesting systems that do not naturally occur in plants. Photosynthesis uses wavelengths from 400 to 700 nm, less than half of the solar spectrum (Figure 2). It is thought that extending the photosynthetically usable light to 750 nm could result in an increase in the number of available photons by 19% [22]. Absorbing light in the far red could potentially be accomplished by inserting cyanobacterial genes encoding the enzymes responsible for chlorophyll *d* or chlorophyll *f* synthesis into plants [23] (Figure 2). Introducing alternative light-harvesting complexes that absorb better in regions where chlorophyll is less efficient, like cyanobacterial phycobilisome components [24] or engineered fluorescence proteins [25], is also a possibility.

## Re-engineering the photosystems

Other interesting proposals consist of engineering crops to express a red-shifted PSI-like reaction centre from anoxygenic photosynthetic bacteria containing bacteriochlorophyll with an absorption maximum at 1100 nm [26]. Another version of this proposal suggested that a crop could be engineered to express an entire red-shifted cyanobacterial PSII complex optimized to use chlorophyll *d* in tandem with a PSII-like anoxygenic reaction centre using bacteriochlorophyll *b* [7]. Even more complex yet, is the possibility of engineering plants that could express different photosystems and antennae in different parts of the canopy or dynamically in response to fluctuating light conditions. However, it has been pointed out that red-shifted photosystems might have less energy available to provide the protective energy barrier needed in PSII to slow down electron transfer back-reactions, resulting in more damage under a variable environment [9,27].

These more radical approaches are still on the drawing board and will require not only the introduction of novel pigment synthesis pathways, but also the introduction of foreign photosystem components. In practical terms, it would need the simultaneous manipulation of the nuclear and chloroplast genomes as different components of the photosynthetic machinery are encoded by different genomes. Nevertheless, naturally occurring cyanobacteria could provide the inspiration for the ever-more complex engineering required to develop a crop with a completely remodelled photosynthetic machinery, as many strains have the capacity to fine-tune photosynthesis depending on the environmental circumstances. For example, the cyanobacterium *Chroococcidiopsis thermalis* PCC 7203 encodes six distinct D1 subunits that can be differentially expressed to suit the environment [28,29]. One of the six is a relatively divergent type of D1 subunit (ChlF) that catalyses the final step of chlorophyll *f* synthesis to permit growth in far-red light [23]. The other copies of D1 might be expressed under low or high-light conditions to optimize the function of the PSII complex as observed in other cyanobacteria [30,31]. What is more, *C. thermalis* encodes two additional D2, two distinct CP47 and four distinct CP43 subunits of PSII, and also encodes four alternative PsaB core subunits of PSI and two distinct PsaA [29], which in theory could result in the expression of eight distinct forms of PSI with different properties. How and when these different types of photosystem are expressed is currently unknown as are their biophysical and biochemical characteristics.

## Improving photoprotection in a fluctuating environment

**Figure 5:**
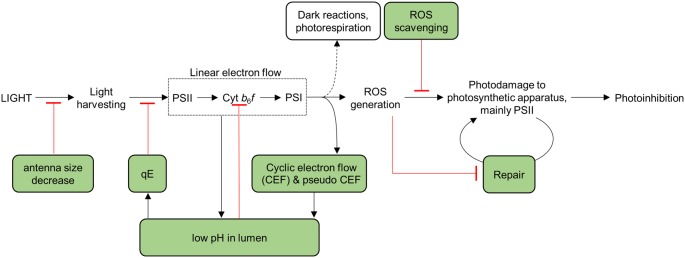
Relationship between photosynthetic electron flow, photodamage and photoprotection The green boxes indicate photoprotective mechanisms. The black arrow indicates causal link while red line indicates negative effect.

In the field, the intensity of light (light quantity) and the spectral profile (light quality) changes through the day, for instance from a passing cloud or a leaf jiggled by the wind, and can vary quickly and unpredictably [32,33]. Variations in light intensity, in particular during conditions of abiotic stress that inhibit carbon fixation, can lead to excess excitation resulting in the formation of excited triplet chlorophyll states that can react with O_2_ to produce damaging reactive oxygen species (ROS) [34–36]. To prevent or mitigate damage, many different protective mechanisms operate at different timescales and at different points of the excitation and electron transfer processes to maximize efficient light use and minimize damage [37]. These include modification of the energetics of the cofactors in PSII to prevent dangerous back-reactions [38], the activation of non-photochemical quenching (NPQ) mechanisms to dissipate excess excitation in the light-harvesting complexes as heat [39,40], pH-induced slowing of electron flow at the level of the Cyt *b_6_f* complex [41] (Figure 5) and the redirection of electron flow via protective alternative pathways such as the plastid terminal oxidase (PTOX) [42] (Figure 1).

A mismatch between the speed of response of the regulatory processes to changes in illumination means that performance of plants in the field could be less than optimal [43]. Therefore, and in the words of Murchie and Niyogi [44], “*An exciting possibility is that manipulating photoprotective pathways is a means to enhance both stress resistance and photosynthetic productivity of crop plants*.”

Along these lines, Kromdijk and colleagues [45] have developed a line of tobacco that overexpresses two enzymes, violaxanthin de-epoxidase and zeaxanthin epoxidase, to boost the xanthophyll cycle needed for NPQ, plus the PsbS subunit which, when triggered by low lumenal pH, enhances the rate of formation of the energy-dependent qE component of NPQ [39]. These alterations allowed the plant to bounce back from a dissipative heat-producing state into a productive light-harvesting state at a faster rate than the wild-type, resulting in an increase in dry-weight biomass of up to 20%, along with increases in leaf area and plant height in field experiments.

Similarly, it might be possible to accelerate the state transition response in which light-harvesting complexes migrate between the laterally segregated PSII and PSI complexes in the thylakoid membrane to maintain balanced excitation of the two photosystems under fluctuating light. The mechanism involves phosphorylation of light-harvesting complex (LHC) proteins by the STN7 kinase, under the control of the redox state of the plastoquinone pool, to form so-called ‘state 2’ in which excitation is redirected to PSI. The dephosphorylation of LHC to reform ‘state 1’ is catalyzed by a constitutively active phosphatase [46].

Flavodiiron (Flv) proteins protect against photodamage under fluctuating light by redirecting electrons from NADPH to oxygen thereby avoiding over-reduction in the electron transfer chain and preventing PSI-mediated superoxide formation [47]. Flv proteins are ubiquitous in all cyanobacteria and are found in many plants but were naturally lost in the ancestor to all flowering plants [48]. Re-introduction of Flv proteins into *Arabidopsis* [49] and tobacco [50] had no effects on photosynthesis under steady light conditions but sped up recovery of photochemistry under fluctuating light. It is hypothesized that the introduction of Flv proteins in crop plants could result in greater yields or biomass in the field [49] and experiments are underway to test this [50]. One complicating factor is that the introduction of Flv proteins might perturb the production of H_2_O_2_ in the chloroplast, which is used a signalling molecule to regulate nuclear gene expression [51].

The synthesis of ATP is driven by the proton motive force (*pmf*), which is dependent on the proton gradient (ΔpH) and the membrane potential (ΔΨ) generated across the thylakoid membrane by the movement of protons and other ions. Under steady-state conditions the proton gradient is considered the dominant component of *pmf* [52] consistent with its crucial role in regulating photosynthetic electron flow. Acidification of the lumen caused by a shift to high-light irradiance activates qE quenching to dissipate excess light energy [39] and slows down linear electron flow at the level of the Cyt *b_6_f* complex thereby protecting PSI from damage [41] (Figure 5).

On the other hand, under fluctuating light, spikes in the membrane potential would act to destabilize the charge-separated states in PSII potentially increasing the rates of back-reaction and singlet oxygen formation and irreversible damage to PSII [53]. Ion fluxes across the membrane, mediated by a large number of channels and transporters (Figure 1), are key to the regulation of the membrane potential and pH gradient under fluctuating light [54]. For example, *Arabidopsis* mutants with a silenced two-pore K^+^ channel (TPK3) in the thylakoid membrane displayed reduced NPQ and enhanced ΔΨ, which resulted in PSII damage under high-light conditions [55]. Another study on the function of a K^+^ efflux antiporter (KEA3) in the thylakoid showed that it was needed for efficient deactivation of NPQ and recovery of productive photochemistry in a transition from high-light to low-light [56]. Davis et al. [57] using computational modelling hypothesized that accelerating the rates of counter-ion fluxes across the thylakoid could lead to improved photosynthetic performance under fluctuating light. This effect could potentially be achieved by overexpressing specific transporters in the membrane, which could translate in more efficient activation and deactivation of non-photochemical qE quenching and thus decreasing ROS production, resulting in long-term higher productivity [57].

The most extreme forms of photoprotection are not found in flowering plants but in strains of the green alga *Chlorella* and diatoms. For example, *Chlorella ohadii* can grow in extreme high light, double the maximum solar intensity, with growth rates that surpass that of any other phototroph and with minimal photodamage [58,59]. It has a naturally reduced antenna size and it is thought that PSII can enter a protective state where electrons are cycled within the complex and thus linear electron flow is blocked. Remarkably, under these extreme conditions, 90% of the electron flow is cyclic while only 10% is linear [58]. Similar to *Chlorella*, the diatom *Phaeodactylum tricornutum* also shows resistance to photodamage using cyclic electron flow around PSII, which it can activate in 1 s. In addition, it can also activate NPQ to a larger and more rapid extent than in most organisms [60]. Needless to say, diatoms are one of the most successful organisms capable of oxygenic photosynthesis accounting for up to 45% of oceanic primary production making them more productive than all the tropical rain forests [61]. One can wonder if such success can be in part due to their superior photoprotective mechanisms and if any such traits can be engineered in crop plants to derive yield advantages under particular environmental conditions. It is worth pointing out that the exact molecular mechanism of cyclic electron flow around PSII is not completely understood, but is likely to involve electron transfer via Cyt *b-559* and a carotenoid bound to the D2 subunit [62]. One obvious next step is to express the core subunits of PSII from these strains in a model plant.

Despite the existence of various layers of photoprotection (Figure 5), PSII in most plants is still damaged irreversibly by light [63]. However, this is mitigated by the operation of an elaborate repair cycle involving partial disassembly of the damaged complex, replacement of the damaged subunit, usually the D1 subunit, and reassembly of the active complex [64]. PSII repair, which itself is sensitive to oxidative stress, involves a number of accessory factors that are important not only for maintenance of PSII activity under continuous high light but also under fluctuating light [65,66]. PSII repair in chloroplasts involves the migration of the damaged PSII complex in the appressed membranes of the grana to the repair apparatus located in the non-appressed stromal membranes. Future work is required to assess whether PSII repair can be improved through overexpression of the proteases, kinases, phosphatases and assembly factors involved in this process [65].

## Producing more ATP

ATP is a vital output of the light reactions as it is needed for fixing CO_2_ and for providing energy to the cell to combat abiotic stress. The amount of ATP produced per NADPH is determined in large part by the proportion of cyclic electron flow around PSI compared with linear electron flow. Surprisingly, the molecular details and regulation of the complexes involved in cyclic electron flow (the PGR5/PGRL1 and NDH pathways, Figure 1) are still poorly understood [67]. Such knowledge will be invaluable in designing plants that are able to thrive under conditions demanding elevated levels of ATP production.

The ATP synthase itself is also an interesting target. The number of c subunits in the c ring of the F_o_ complex determines the stoichiometry of ATP produced per H^+^ translocated through the complex [68]. In the case of the chloroplast ATP synthase the ring consists of 14 subunits [69] compared with 8 found in the bovine enzyme [70]. Re-engineering the ATP synthase to have a smaller ring would automatically boost the amount of ATP produced per NADPH in linear electron flow and would be particularly advantageous under conditions of constant illumination.

## Concluding remarks: super-crops

It is widely recognized that the efficiency of photosynthesis is a trade-off between energy invested in growth and energy wasted or invested in protection and repair [24,57,71,72]. As a consequence, crops of the future will be tailor made for maximum productivity in the particular environment where they will be grown. In that way, a crop destined to be grown in a greenhouse with tightly controlled environmental conditions can be engineered to forgo most photoprotective mechanisms (such as the qE component of NPQ) in favour of maximum yield, while a crop designed to grow in dry high-salinity soil may incorporate a suite of fast acting regulatory, protective and repair mechanisms that help the plant optimize yield.

Current efforts to engineer crops with better photosynthetic performance consist of adding, removing or modifying the expression of a few genes, and these rather simple approaches seem to show some promise as exemplified above. However, the super-crops of the future are likely to be extensively genetically modified. They may feature antenna and photosystem components from multiple sources, some might be ancestrally reconstructed, while others may have been developed by extensive directed evolution. The design of a smart chloroplast with a multiplicity of photosystem core proteins that can be differentially expressed with environmental fluctuations may be a possibility. Such a system may be complemented with enhanced carbon assimilation processes and redesigned metabolic pathways leading to grain or fruit with particular nutritional content. Leaf shape, plant size and rates of growth may also be altered to match regions of the world with distinct seasonal and diurnal variations in light availability. To top it off, it is plausible that interactions between the crop and their complex microbiome can also be manipulated to enhance plant health or completely do without human-made fertilizers [73,74].

## Summary

- Genetic engineering of the light reactions of photosynthesis could potentially lead to improvements in crop yields.
- The efficiency of photosynthesis is a balance between the energy invested in growth and the energy used or expended in protection and repair mechanisms.
- A promising strategy for improving photosynthesis is the design of crops that can quickly bounce back from photoinhibited states and respond faster to fluctuating light conditions.
- Many appealing proposals to improve photosynthesis in plants are still at a very early stage of conception and will require extensive genetic engineering of the nuclear and chloroplast genomes.

## Abbreviations

Flv: flavodiiron
LHC: light-harvesting complex
NDH: NADH dehydrogenase-like
NPQ: non-photochemical quenching
PGR5: proton gradient regulation 5
PGRL1: PGR5-like protein 1
*pmf*: proton motive force
PSI: photosystem I
PSII: photosystem II
ROS: reactive oxygen species
